# Implementation of Tuberculosis Intensive Case Finding, Isoniazid Preventive Therapy, and Infection Control ("Three I's") and HIV-Tuberculosis Service Integration in Lower Income Countries

**DOI:** 10.1371/journal.pone.0153243

**Published:** 2016-04-13

**Authors:** M. Katherine Charles, Mary Lou Lindegren, C. William Wester, Meridith Blevins, Timothy R. Sterling, Nguyen Thi Dung, Jean Claude Dusingize, Divine Avit-Edi, Nicolas Durier, Barbara Castelnuovo, Gertrude Nakigozi, Claudia P. Cortes, Marie Ballif, Lukas Fenner, International epidemiology Databases to Evaluate AIDS (IeDEA) Collaboration

**Affiliations:** 1 Vanderbilt Univ. School of Medicine, Vanderbilt Institute for Global Health (VIGH), Nashville, Tennessee, United States of America; 2 National Hospital of Tropical Diseases, Hanoi, Vietnam; 3 Women's Equity in Access to Care & Treatment, Kigali, Rwanda; 4 MTCTPlus, Abidjan, Cote d’Ivoire; 5 TREAT Asia, amfAR–The Foundation for AIDS Research, Bangkok, Thailand; 6 Infectious Disease Institute, Kampala, Uganda; 7 Rakai Health Sciences Program, Kalisizo, Uganda; 8 University of Chile School of Medicine, Santiago, Chile; 9 Institute of Social and Preventive Medicine, University of Bern, Bern, Switzerland; 10 Swiss Tropical and Public Health Institute, Basel, Switzerland; 11 University of Basel, Basel, Switzerland; Kings College London, UNITED KINGDOM

## Abstract

**Setting:**

World Health Organization advocates for integration of HIV-tuberculosis (TB) services and recommends intensive case finding (ICF), isoniazid preventive therapy (IPT), and infection control (“Three I’s”) for TB prevention and control among persons living with HIV.

**Objective:**

To assess the implementation of the “Three I’s” of TB-control at HIV treatment sites in lower income countries.

**Design:**

Survey conducted between March-July, 2012 at 47 sites in 26 countries: 6 (13%) Asia Pacific, 7 (15%), Caribbean, Central and South America, 5 (10%) Central Africa, 8 (17%) East Africa, 14 (30%) Southern Africa, and 7 (15%) West Africa.

**Results:**

ICF using symptom-based screening was performed at 38% of sites; 45% of sites used symptom-screening plus additional diagnostics. IPT at enrollment or ART initiation was implemented in only 17% of sites, with 9% of sites providing IPT to tuberculin-skin-test positive patients. Infection control measures varied: 62% of sites separated smear-positive patients, and healthcare workers used masks at 57% of sites. Only 12 (26%) sites integrated HIV-TB services. Integration was not associated with implementation of TB prevention measures except for IPT provision at enrollment (42% integrated vs. 9% non-integrated; p = 0.03).

**Conclusions:**

Implementation of TB screening, IPT provision, and infection control measures was low and variable across regional HIV treatment sites, regardless of integration status.

## Introduction

In countries with high burdens of HIV and tuberculosis (TB), the World Health Organization (WHO) recommends 12 collaborative HIV-TB activities as a part of core prevention, care, and treatment services.[1] These activities have three distinct objectives: improving mechanisms for integrated delivery of TB and HIV services, reducing the burden of HIV among people with presumptive and diagnosed TB, and reducing the burden of TB among people living with HIV and initiating early antiretroviral therapy. The “Three I’s,” Intensive case finding (ICF), Isoniazid preventive therapy (IPT), and Infection control, are the mainstay of guidelines for reducing TB in persons living with HIV.[1–4] Yet a growing body of data suggests that implementation of the “Three I’s” is suboptimal at HIV care and treatment sites, particularly at those without on-site or integrated TB treatment services.[5–8]

WHO encourages the integration of HIV and TB services, as studies conducted at integrated sites have demonstrated improvements in TB treatment outcomes, including the timeliness of ART initiation.[9–11] Prior studies have demonstrated the feasibility of integrating HIV-TB services, even in rural settings,[10, 12–14] though there lacks consensus on the best model for integration.[9, 13] This study investigated the implementation of the “Three I’s” at HIV care and treatment sites in lower income countries within the International epidemiologic Databases to Evaluate AIDS (IeDEA) consortium, examining how the integration of HIV-TB services and other healthcare facility characteristics may be associated with the implementation of the “Three I’s” to reduce the burden of TB among persons living with HIV.

## Methods

### Study setting

The present study is a sub-study of a larger survey of TB diagnosis and screening approaches throughout the IeDEA consortium.[15] The IeDEA consortium is an international research network of HIV care and treatment sites located in seven regions globally: Asia/Pacific, the Caribbean, Central and South America (CCASAnet), North America, Central Africa, East Africa, Southern Africa, and West Africa. A total of 71 sites in low- and middle-income countries were invited to join the larger study, and 58 sites (81.7%) in 26 countries participated. We included the 47 sites that treat adults aged 16 years and older, excluding 11 pediatric sites that treated children only. Participating sites were surveyed between March 1 and July 1, 2012.

### Data collection

Surveys were developed within the IeDEA TB working group with input from advisory board members from each represented region.[15] Surveys were written first in English and translated to French and Spanish when appropriate. Pilot testing was conducted in all languages. The survey was completed by medical staff or local data managers, and data were collected and managed electronically in a secure REDCap database (Research Electronic Data Capture; https://project-redcap.org). We collected site-level characteristics related to integration of HIV-TB services, such as the level of care, setting (urban vs. rural), number of HIV patients actively cared for, number of TB cases seen, as well as the availability of preventive, screening, diagnostic, and treatment services for TB.

### Outcome measures

ICF was measured via: (1) use of a standard four-symptom screening tool (cough, fever, weight loss, and night sweats) at enrollment and follow-up visits, and (2) any additional household interventions or contact tracing. IPT use was measured by assessing the circumstances for which IPT was given (for all adults at ART start or enrollment vs. TST-positive only) and the duration of use. Infection control was assessed through the use of the specific administrative procedures, environmental controls, and personal protection for healthcare workers. The turn-around time for TB diagnosis was measured as number of days between the initial clinic visit of a symptomatic patient and the date of TB treatment initiation.

Site-level integration was determined by TB screening practices and location of TB clinical service delivery, based upon WHO recommended activities and prior published studies. [5, 6, 9, 13, 14] A site with integrated HIV-TB services met all of the following criteria: (1) persons living with HIV are actively screened for TB at enrollment using symptom screening; (2) TB and HIV clinical services are located in the same facility, under the same roof, or available with same day appointments; and (3) facilities have a specialized clinic/ward on site with dedicated staff for patients with TB.

### Statistical analysis

Descriptive statistics summarize the implementation of the “Three I’s” and service integration by region. We used chi-squared and Wilcoxon rank sum tests to test for associations between site characteristics, including the integration of HIV-TB services, and the implementation of the “Three I’s.” Analyses were performed using R-software version 2.11.1 (www.r-project.org).

### Ethics statement

Data were collected through IeDEA cohorts. Ethics committees and/or institutional review boards in all host countries approved the collection and transfer of anonymous data; a complete list is found in S2 File. Written informed consent was obtained when requested per local regulations. In addition, the Vanderbilt Human Research Protection Program’s Health Sciences Committee, Nashville, Tennessee (USA), the Ethics Committee of the University of Bern (Switzerland), and the University of Cape Town (South Africa) approved the analyses for this specific project.

## Results

### Program characteristics

Data were collected from 47 ART sites treating HIV-infected adults in six IeDEA regions: 6 (13%) Asia-Pacific, 7 (15%) CCASAnet, 5 (11%) Central Africa, 8 (17%) East Africa, 14 (30%) Southern Africa, and 7 (15%) West Africa. The majority of treatment sites were in urban settings (81%), 7 (15%) peri-urban and 2 (4%) rural. During the study period, 251,377 ART patients were in care across the 6 regions (range: 8,861 in Asia/Pacific to 103,954 in East Africa). By ART site reports, 17,748 new cases of TB were detected during 2011 (range: 296 in Asia-Pacific to 12,378 in Southern Africa). Tables and figures describing program characteristics by region have been previously published.[15]

### Intensified case finding (ICF)

At the time of enrollment into HIV care, 18 (38%) sites screened using clinical symptom screening only (cough, weight loss, fever, and/or night sweats), 21 (45%) sites reported utilizing symptom-based screening plus other diagnostics, while 8 (17%) sites relied on clinical suspicion only (Table 1). Twenty-six (55%) sites report using four-symptom screening for TB among patients living with HIV at follow-up. Less than half of sites reported tuberculin skin testing (TST) on site (45%), though Asia-Pacific and CCASAnet reported 83% and 86%, respectively.

**Table 1:** Implementation of the “Three I’s”* and integration of HIV-TB services in 47 HIV care and treatment facilities treating adults in lower-income countries within the IeDEA collaboration, overall and by IeDEA region.

	Asia/ Pacific	CCASAnet	Central Africa	East Africa	Southern Africa	West Africa	TOTAL
	(n = 6)	(n = 7)	(n = 5)	(n = 8)	(n = 14)	(n = 7)	(n = 47)
**Intensified case finding (ICF)**							
TB screening at enrollment into HIV care, n (%)							
Symptom screening	1 (17%)	0 (0%)	2 (40%)	4 (50%)	7 (50%)	4 (57%)	18 (38%)
Symptom screening plus additional diagnostics	4 (67%)	5 (71%)	0 (0%)	3 (38%)	7 (50%)	2 (29%)	21 (45%)
Clinical suspicion	1 (17%)	2 (29%)	3 (60%)	1 (12%)	0 (0%)	1 (14%)	8 (17%)
Screening algorithm during follow-up include the following symptoms and/or diagnostic tests, n (%)							
Four-symptom screening†	2 (33%)	1 (14%)	3 (60%)	6 (75%)	10 (71%)	4 (57%)	26 (55%)
Cough (any duration)	3 (50%)	1 (14%)	3 (60%)	6 (75%)	10 (71%)	4 (57%)	27 (57%)
Fever (any duration)	3 (50%)	2 (29%)	3 (60%)	7 (88%)	10 (71%)	5 (71%)	30 (64%)
Night sweats	2 (33%)	2 (29%)	3 (60%)	7 (88%)	10 (71%)	6 (86%)	30 (64%)
Weight loss	3 (50%)	2 (29%)	3 (60%)	7 (88%)	10 (71%)	6 (86%)	31 (66%)
Contact history with a TB case in family	3 (50%)	2 (29%)	3 (60%)	4 (50%)	7 (50%)	6 (86%)	25 (53%)
Sputum AFB smear	3 (50%)	2 (29%)	2 (40%)	3 (38%)	5 (36%)	4 (57%)	19 (40%)
Induced sputum	1 (17%)	1 (14%)	0 (0%)	0 (0%)	0 (0%)	0 (0%)	2 (4%)
Gastric lavage	0 (0%)	1 (14%)	0 (0%)	0 (0%)	0 (0%)	0 (0%)	1 (2%)
Biopsy	2 (33%)	2 (29%)	1 (20%)	2 (25%)	1 (7%)	0 (0%)	8 (17%)
Chest radiography	3 (50%)	2 (29%)	2 (40%)	4 (50%)	5 (36%)	4 (57%)	20 (43%)
TB culture	1 (17%)	0 (0%)	1 (20%)	0 (0%)	1 (7%)	1 (14%)	4 (9%)
Other	0 (0%)	0 (0%)	0 (0%)	1 (12%)	1 (7%)	1 (14%)	3 (6%)
Specific intensified case finding program, n (%)	2 (33%)	2 (29%)	3 (60%)	6 (75%)	7 (50%)	3 (43%)	23 (49%)
Household intervention with contact tracing, n (%)	0 (0%)	1 (14%)	1 (20%)	3 (38%)	6 (43%)	1 (14%)	12 (26%)
**Isoniazid preventive therapy (IPT)**							
IPT for all adults (where active TB ruled out) at enrollment or time of ART start, n (%)	1 (17%)	2 (29%)	0 (0%)	1 (12%)	3 (21%)	1 (14%)	8 (17%)
Only if TST-positive, n (%)	1 (17%)	3 (43%)	0 (0%)	0 (0%)	0 (0%)	0 (0%)	4 (9%)
Duration of IPT, n (%)‡							
6 months	1 (17%)	0 (0%)	0 (0%)	0 (0%)	3 (21%)	1 (14%)	5 (11%)
9–12 months	1 (17%)	4 (57%)	0 (0%)	1 (12%)	0 (0%)	0 (0%)	6 (13%)
Lifetime	0 (0%)	1 (14%)	0 (0%)	0 (0%)	0 (0%)	0 (0%)	1 (2%)
None	4 (67%)	2 (29%)	5 (100%)	7 (88%)	10 (71%)	6 (86%)	34 (72%)
**Infection control measures**							
Separation of sputum smear-positive TB patients and HIV-positive patients, n (%)	5 (83%)	4 (57%)	3 (60%)	5 (62%)	9 (64%)	3 (43%)	29 (62%)
Separate waiting rooms and emergency wards for "coughing" patients, n (%)	2 (33%)	2 (29%)	1 (20%)	4 (50%)	4 (29%)	1 (14%)	14 (30%)
Natural air exchange by dedicated windows, n (%)							
Optimized natural ventilation	3 (50%)	3 (43%)	2 (40%)	4 (50%)	8 (57%)	1 (14%)	21 (45%)
Natural ventilation, but not optimized	1 (17%)	4 (57%)	3 (60%)	4 (50%)	6 (43%)	6 (86%)	24 (51%)
No natural ventilation	2 (33%)	0 (0%)	0 (0%)	0 (0%)	0 (0%)	0 (0%)	2 (4%)
Operated and maintained ventilators, n (%)	3 (50%)	5 (71%)	2 (40%)	1 (12%)	4 (29%)	2 (29%)	17 (36%)
TB screening for medical staff working with TB patients, n (%)	5 (83%)	5 (71%)	0 (0%)	3 (38%)	3 (21%)	1 (14%)	17 (36%)
Staff wear masks in close contact to TB patients, n (%)	5 (83%)	6 (86%)	2 (40%)	3 (38%)	7 (50%)	4 (57%)	27 (57%)
Staff offered no specified TB protection, n (%)	1 (17%)	1 (14%)	3 (60%)	1 (12%)	7 (50%)	3 (43%)	16 (34%)
Turn-around time for TB diagnosis, median days (IQR)§							
Smear-positive patients	7 (5–7)	7 (2–7)	4 (4–7)	2 (1–2)	3 (2–5)	5 (4–7)	4 (2–7)
Smear-negative patients	6 (2–17)	14 (5–18)	14 (14–15)	5 (2–6)	7 (3–14)	15 (10–15)	7 (4–14)
**TB services**							
TB skin test available on site, n (%)	5 (83%)	6 (86%)	2 (40%)	1 (12%)	4 (29%)	3 (43%)	21 (45%)
TB clinic location, n (%)							
Same facility or same day	1 (17%)	4 (57%)	2 (40%)	4 (50%)	7 (50%)	3 (43%)	21 (45%)
Cross referral between HIV-TB service points	4 (67%)	2 (29%)	1 (20%)	3 (38%)	5 (36%)	3 (43%)	18 (38%)
Provision of HIV-TB services under same roof	1 (17%)	1 (14%)	1 (20%)	1 (12%)	2 (14%)	1 (14%)	7 (15%)
None of these models	0 (0%)	0 (0%)	1 (20%)	0 (0%)	0 (0%)	0 (0%)	1 (2%)
Availability of a specialized clinic/ward on site with dedicated staff for TB patients, n (%)							
Yes, on site	3 (50%)	6 (86%)	4 (80%)	6 (75%)	7 (50%)	2 (29%)	28 (60%)
No, but available off site (referral)	2 (33%)	1 (14%)	0 (0%)	1 (12%)	2 (14%)	4 (57%)	10 (21%)
Not available	1 (17%)	0 (0%)	1 (20%)	1 (12%)	5 (36%)	1 (14%)	9 (19%)
**ART provision**, n (%)							
All HIV-infected patients	4 (67%)	6 (86%)	3 (60%)	6 (75%)	10 (71%)	5 (71%)	34 (72%)
<200 CD4+ cell count	0 (0%)	0 (0%)	0 (0%)	0 (0%)	0 (0%)	1 (14%)	1 (2%)
<350 CD4+ cell count	2 (33%)	1 (14%)	2 (40%)	2 (25%)	4 (29%)	1 (14%)	12 (26%)
**Integration of HIV-TB services**, n (%)‖							
Integrated	0 (0%)	3 (43%)	0 (0%)	4 (50%)	4 (29%)	1 (14%)	12 (26%)
Not integrated	6 (100%)	4 (57%)	5 (100%)	4 (50%)	10 (71%)	6 (86%)	35 (74%)

*“Three I’s” defined as (1) intensified case finding, (2) IPT, (3) infection control

^†^Four symptom screening includes: cough, fever, night sweat, and weight loss

^‡^One site in Southern Africa reported unspecified “other” conditions for and duration of IPT use

^§^Continuous variables are reported as median number of days (interquartile range)

^‖^Integration clinics met the following criteria: (1) HIV patients screened for TB at enrollment using at least symptom screening; (2) TB clinic was located in the same facility/same day or under the same roof; and (3) facilities had specialized clinic/ward on site with dedicated staff for TB patients

CCASAnet = Caribbean, Central and South America; TB = tuberculosis; ART = combination antiretroviral therapy; TST = tuberculin skin testing; IQR = interquartile range

Twenty-three (49%) programs reported having a specific program for ICF, ranging from 29% in CCASAnet to 75% in East Africa (Table 1). Twelve (26%) sites reported conducting household assessments (ranging from 29% in CCASAnet to 75% in East Africa) as their primary means of contact tracing. Four sites (9%) reported performing door-to-door screening.

### Isoniazid preventive therapy (IPT)

IPT was prescribed to all adults (after active TB disease was ruled out) at the time of enrollment or at ART initiation in 17% of sites, which ranged from no use of IPT in Central African sites to 29% at CCASAnet sites. Use of IPT for patients with a positive TST only was reported by 9% of sites, with the highest in CCASAnet (43%). Among sites providing IPT, 11% of sites prescribed it for 6 months, 13% for 9–12 months, and 2% lifelong.

### Infection control

Separation of patients with sputum smear-positive TB and persons living with HIV on the wards, the emergency room, and/or outpatient clinics was a self-reported standard practice at 29 sites (62%), ranging from 43% in West Africa to 83% in Asia-Pacific. Separate waiting rooms or emergency departments for coughing patients were not available in the majority (70%) of sites. The median turn-around time between clinic visit of a symptomatic patient and TB treatment initiation was 4 days for smear-positive patients (interquartile range (IQR) 2–7 days) and 7 days for smear-negative patients (IQR 4–14). Environmental control measures included “optimized” windows for airflow by size and location (on opposing walls) (45%), natural ventilation but not “optimized” (51%), and no natural ventilation measures reported (4%).

Operated and maintained ventilators were present in 36% of sites. Personal protection for healthcare workers was low; only 17 sites (36%) provided TB screening for medical staff working with TB patients, ranging from 83% of sites in Asia-Pacific to no sites in Central Africa. Only 27 sites (57%) reported staff routinely wore masks when in close contact with TB patients; the remaining sites either did not report or reported that healthcare personnel wore no protective masks. At least one site in each region reported that staff was offered no specific TB protection, ranging from 12% in East Africa to 60% in Central Africa.

### Integration of HIV-TB services

HIV-TB services were located within the same facility or same day services at 45% of sites. Specialized clinics with dedicated staff for TB patients were available onsite for 60% of facilities; 21% reported dedicated staff off site, and 19% reported no specialized HIV-TB clinics on or off site.

Only 12 clinics (26%) met all three of our criteria for an integrated HIV-TB clinic; three were located in CCASAnet, four in East Africa, four in Southern Africa, and one in West Africa. Neither Asia-Pacific nor Central Africa reported any clinic meeting these criteria. We did not detect significant differences in program characteristics between integrated and non-integrated sites (Table 1). Participating sites were predominantly urban regardless of whether they were integrated (83%) or not (80%). Of the integrated sites, 17% were primary, 33% were secondary, and 50% were tertiary, similar to non-integrated sites (primary 27%, secondary 11%, and tertiary 52%). The majority of integrated (58%) and non-integrated sites (71%) treated both adults and children. Despite only 12 of 47 sites being integrated, integrated sites collectively managed a higher patient volume (134,300 patients) than non-integrated sites (117,047 patients).

### Integration of HIV-TB services and implementation of the Three I’s

A greater proportion of integrated compared to non-integrated sites reported specific ICF programs (67% vs. 43%); and 33% of integrated vs. 23% on non-integrated sites reported specific household interventions with contact tracing (Table 2). However, these differences were not statistically significant (p = 0.27, p = 0.74, respectively). There was no statistically significant difference in integrated vs. non-integrated sites using four-symptom screening for patients living with HIV at follow-up (50% vs. 57%, p = .67).

**Table 2:** Program Characteristics and Implementation of Three I’s by HIV-TB service integration status in 47 ART programs treating adults in lower income countries.

	Integrated	Not integrated	TOTAL	P-value
	(n = 12)	(n = 35)	(n = 47)	
**IeDEA region, n (%)**				0.16
Asia-Pacific	0 (0%)	6 (17%)	6 (13%)	
CCASAnet	3 (25%)	4 (11%)	7 (15%)	
Central Africa	0 (0%)	5 (14%)	5 (11%)	
East Africa	4 (33%)	4 (11%)	8 (17%)	
Southern Africa	4 (33%)	10 (29%)	14 (30%)	
West Africa	1 (8%)	6 (17%)	7 (15%)	
Number of countries, n*	10	22	26	
**Setting, n (%)**				0.16
Urban	10 (83%)	28 (80%)	38 (81%)	
Peri-urban	1 (8%)	6 (17%)	7 (15%)	
Rural	1 (8%)	1 (3%)	2 (4%)	
**Level of care, n (%)**				0.58
Primary	2 (17%)	13 (27%)	15 (32%)	
Secondary	4 (33%)	4 (11%)	8 (17%)	
Tertiary	6 (50%)	18 (52%)	24 (51%)	
**Patient population, n (%)**				0.40
Adults and children	7 (58%)	25 (71%)	32 (68%)	
Adults only	5 (42%)	10 (29%)	15 (32%)	
**ART provision, n (%)**				0.84
All HIV-infected patients	9 (75%)	25 (71%)	34 (72%)	
<200 CD4	0 (0%)	1 (3%)	1 (2%)	
<350 CD4	3 (25%)	9 (26%)	12 (26%)	
**Intensified case finding**				
Screening algorithm during follow-up include the following symptoms and/or diagnostic tests, n (%)				
Four symptom screening†	6 (50%)	20 (57%)	26 (55%)	0.67
Cough (any duration)	6 (50%)	21 (60%)	27 (57%)	0.79
Fever (any duration)	8 (67%)	22 (63%)	30 (64%)	0.99
Night sweats	8 (67%)	22 (63%)	30 (64%)	0.99
Weight loss	8 (67%)	23 (66%)	31 (66%)	0.99
Contact history with a TB case in family	4 (33%)	21 (60%)	25 (53%)	0.21
Sputum AFB smear	5 (42%)	14 (40%)	19 (40%)	0.99
Induced sputum	0 (0%)	2 (6%)	2 (4%)	0.99
Gastric lavage	1 (8%)	0 (0%)	1 (2%)	0.57
Biopsy	3 (25%)	5 (14%)	8 (17%)	0.68
Chest radiography	6 (50%)	14 (40%)	20 (43%)	0.79
TB culture	1 (8%)	3 (9%)	4 (9%)	0.99
Other	1 (8%)	2 (6%)	3 (6%)	0.99
Specific intensified case finding program, n (%)	8 (67%)	15 (43%)	23 (49%)	0.28
Household intervention with contact tracing, n (%)	4 (33%)	8 (23%)	12 (26%)	0.74
**Isoniazid preventive therapy (IPT)**				
IPT available for all adults at enrollment or at ART start	5 (42%)	3 (9%)	8 (17%)	**0.029**
TST-positive only	1 (8%)	3 (9%)	4 (9%)	0.99
Duration of IPT, n (%)‡				0.20
6 months	2 (17%)	3 (9%)	5 (11%)	
9–12 months	3 (25%)	3 (9%)	6 (13%)	
Lifetime	1 (8%)	0 (0%)	1 (2%)	
Never (not administered)	6 (50%)	29 (82%)	35 (74%)	
**Infection control measures**				
Separation of sputum smear-positive TB patients and HIV-positive patients, n (%)	9 (75%)	20 (57%)	29 (62%)	0.45
Separate waiting rooms and emergency wards for "coughing" patients, n (%)	5 (42%)	9 (26%)	14 (30%)	0.50
Natural air exchange by dedicated windows, n (%)				0.673
Optimized natural ventilation	6 (50%)	15 (43%)	21 (45%)	
Natural ventilation, but not optimized	6 (50%)	18 (51%)	24 (51%)	
No natural ventilation	0 (0%)	2 (6%)	2 (4%)	
Operated and maintained ventilators, n (%)	6 (50%)	11 (31%)	17 (36%)	0.42
TB screening for medical staff working with TB patients, n (%)	5 (42%)	12 (34%)	17 (36%)	0.91
Staff wears masks in close contact to TB patients, n (%)	7 (58%)	20 (57%)	27 (57%)	0.99
Staff offered no specified TB protection, n (%)	3 (25%)	13 (37%)	16 (34%)	0.68
Turn-around time for TB diagnosis, median days (IQR)§				
Smear-positive patients	3 (2, 5)	4 (2, 7)	4 (2, 7)	0.27
Smear-negative patients	10 (3, 14)	7 (4, 14)	7 (4, 14)	0.51

*Chi-squared and rank sum tests of association between integration and site characteristic

^†^Four symptom screening includes: cough, fever, night sweat, and weight loss

^‡^One site in Southern Africa reported unspecified “other” conditions for and duration of IPT use

^§^Continuous variables are reported as medians (interquartile range)

CCASAnet = Caribbean, Central and South America; TB = tuberculosis; ART = antiretroviral therapy; AFB = acid fast bacilli

Treatment with IPT for all adults at either enrollment or ART start was significantly greater in integrated (42%) than non-integrated sites (9%) (p = 0.03), while IPT use for TST-positive patients only was similar for integrated (8%) and non-integrated (9%) sites (p = 0.99). There were no statistically significant differences in the implementation of infection control measures, though integrated sites reported slightly greater implementation compared to non-integrated sites. Integrated sites reported greater separation of sputum smear-positive TB patients and HIV-positive patients (75% vs. 57%, p = 0.45), and greater separation of coughing patients in waiting rooms and emergency wards (42% vs. 26%, p = 0.50). A slightly greater, though not statistically significant proportion of integrated sites reported using optimized ventilation (50%) or operating and maintained ventilators (50%), compared to 43% and 31% of non-integrated sites, respectively (p = 0.67). The median turn-around time for smear-negative patients was longer in integrated (10 days) than non-integrated clinics (7 days), though not statistically significant (p = 0.27).

## Discussion

We assessed the implementation of the “Three I’s” of TB prevention and control among persons living with HIV and the association between HIV-TB service integration and the implementation of these measures. In diverse geographic settings, levels of care, patient populations, disease burdens, and HIV-TB integration models, the implementation of the “Three I’s” was low and variable throughout regions. However, we found that IPT was offered routinely to all patients at enrollment or ART start at 42% of integrated sites versus only 9% of non-integrated sites.

Favorable health outcomes depend on early identification and diagnosis of individuals suspected of having TB and early initiation of treatment.[16] A comprehensive meta-analysis including over 8,000 persons living with HIV found that the absence of current cough, fever, night sweats and weight loss was quite sensitive (79%) for ruling out active TB in persons living with HIV/AIDS with low pretest probability of TB infection.[17] Though WHO recommends ICF using this four-symptom screening algorithm to exclude active TB disease and determine who should be initiated on IPT,[2, 3] we found only one-third of clinics used symptom-screening alone to detect TB in HIV-infected patients. We also found that less than half of sites had specific ICF programs, and only 26% implemented household interventions with contact tracing.

Our finding that IPT provision is low in resource-limited ART treatment sites is consistent with recent studies.[9, 12] Concerns regarding the optimal algorithm for excluding active TB before starting IPT may underlie the low acceptance of WHO recommendations. Providers may be concerned about isoniazid resistance, despite evidence that providing IPT to persons living with HIV does not increase the risk of resistance.[3, 18, 19] Recent studies have also demonstrated that the implementation of IPT is strongly linked to ICF activities at HIV treatment sites.[9, 20] Resource-limited sites with operational difficulties in identifying cases or accessing confirmatory diagnostic tests may have substantial delays in the exclusion of active TB and the initiation of IPT.[7, 15]

Though substantial gaps in the implementation of TB infection control measures were identified, we found that clinics were most likely to (1) separate patients with known HIV from those with known active-TB and (2) use masks among staff. Less than half of sites performed any of the other WHO recommended infection control measures. Previous studies demonstrated that new sites opened as part of scale-up initiatives were no more likely to have implemented all TB infection control measures.[8] If the implementation of infection control measures is fueled by healthcare workers who have motivation to protect themselves, infection control training and strong leadership may be crucial for adherence.[9]

We also found that few sites implemented an integrated model of TB and HIV care. Case studies have demonstrated that successful integration of HIV-TB services is contingent upon a common physical space, jointly trained staff, and constant communication.[6, 21] Our study showed that only 60% of sites had services in the same facility or under the same roof, and only one quarter of all sites also had specialized clinics or ward with dedicated staff who performed TB screening for HIV patients upon enrollment. Sites may implement solitary measures to integrate HIV and TB services; however, partially implemented programs may have limited impact on curbing the dual epidemics.[2] Sites that integrate services under one roof, but take no steps to screen all HIV patients for TB upon enrollment may compromise the quality of care. A better understanding of the opportunities and barriers to HIV-TB service integration, with a thorough characterization of successfully integrated sites is crucial for scaling-up this international recommendation.

There are several limitations to this study. Though our sample is comprehensive in that we included HIV care and treatment sites from seven regions throughout the world, it may not be representative for all programs in the regions based on program heterogeneity. This survey reflects the clinical services provided at the time of data collection, but it addresses neither the consistency nor quality of available services. Survey data is subject to bias, and it is possible that sites over-reported compliance with WHO guidelines. Selection bias may have also influenced our findings, as the 13 sites declining to participate in the survey may have been different than those participating. Due to a small sample of integrated sites, we failed to detect statistically significant differences across TB integration even though some differences appear meaningful. We developed a definition of HIV-TB service integration based on our questionnaire. While there is no universally accepted working definition for an integrated HIV-TB clinic, the testing of less stringent definitions revealed no statistically significant difference in outcomes between integrated and non-integrated clinics.

## Conclusions

This study surveyed diverse lower-income ART centers worldwide, showing poor integration of HIV-TB services and low implementation of the “Three I’s.” The integration of HIV-TB services requires proper prevention and control of TB among persons living with HIV. Without these measures, integration may halt or even reverse progress toward controlling the dual-burden of disease.[22, 23] Greater implementation of the “Three I’s”, many of which are facility-based infection control measures, could have significant public health benefits at both the healthcare worker and individual patient level.

## Supporting Information

S1 File Funding, TB working group, and participating sites. (DOC)

S2 File Ethics statement including a full list of Ethics Committees and/or Institutional Review Boards. (DOC)
