# Supplementary material for: Implementation of Tuberculosis Intensive Case Finding, Isoniazid Preventive Therapy, and Infection Control ("Three I's") and HIV-Tuberculosis Service Integration in Lower Income Countries
Source: PLoS One. 2016 Apr 13;11(4):e0153243. doi: 10.1371/journal.pone.0153243 (PMC4830552; doi:10.1371/journal.pone.0153243)
Supplement: S1 File — (DOC) [file pone.0153243.s001.doc]

**Supplemental File S1**: Funding, TB working group, and participating sites

**Funding**: Research reported in this publication was supported by the National Institute of Allergy and Infectious Diseases (NIAID) of the National Institutes of Health (NIH) under the following grant award numbers: Southern Africa (U01AI069924), Asia-Pacific (U01AI069907), Central Africa (U01A096299), East Africa (U01AI069911), the Caribbean, Central and South America (U01AI069923), West Africa (U01AI069919) and the IeDEA Network Coordinating Center (INCC) at Vanderbilt University (U01AI096186). The content is solely the responsibility of the authors and does not necessarily represent the official views of the National Institutes of Health. The funder had no role in study design, data collection and analysis, decision to publish, or preparation of the manuscript.

**The IeDEA Tuberculosis Working Group (2012)**:

Samuel Ajayi, Kathryn Anastos, Marie Ballif, Jules Bashi, William Bishai, Andrew Boulle, Paula Braitstein, Gabriela Carriquiry, Jane E. Carter, Peter Cegielski, Cleophas Chimbetete, Claudia P.Cortes, Mary-Ann Davies, Lameck Diero, Stephany Duda, Nicolas Durier, Jean Claude Dusingize, Matthias Egger, Tanoh F. Eboua, Lukas Fenner (Chairperson), Adrian Gasser, Elvin Geng, Joachim Charles Gnokori, Laura Hardwicke, Chris Hoffmann, Robin Huebner, Nzali Kancheya, Sasisopin Kiertiburanakul, Peter Kim, Diero Lameck, Valériane Leroy, Charlotte Lewden, Mary Lou Lindegren, Anna Mandalakas, Mhairi Maskew, Rosemary McKaig, Lynne Mofenson, Mireille Mpoudi-Etame, Benson Okwara, Sam Phiri, Wasana Prasitsuebsai, April Petit, Hans Prozesky, Stewart E. Reid, Lorna Renner, Gary Reubenson, Annette Sohn, Timothy Sterling, Quynh Vo, Dana Walker, Firas Wehbe, Christian Wejse, C. William Wester, Carolyn Williams, Robin Wood, Kara Wools-Kaloustian, Zhang Yao, and Evy Yunihastuti.

**Participating sites**:

**The IeDEA Asia-Pacific region**: **The TREAT Asia HIV Observational Database (TAHOD):** FJ Zhang, HX Zhao, and N Han, Beijing Ditan Hospital, Capital Medical University, Beijing, China; TP Merati, DN Wirawan, and F Yuliana, Faculty of Medicine Udayana University & Sanglah Hospital, Bali, Indonesia; R Ditangco, E Uy, and R Bantique, Research Institute for Tropical Medicine, Manila, Philippines; P Phanuphak, K Ruxrungtham, A Avihingsanon, and M Khongphattanayothin, HIV-NAT/Thai Red Cross AIDS Research Centre, Bangkok, Thailand; S Kiertiburanakul†, S Sungkanuparph, and N Sanmeema, Faculty of Medicine, Ramathibodi Hospital, Mahidol University, Bangkok, Thailand; R Chaiwarith, T Sirisanthana, and W Kotarathititum, Research Institute for Health Sciences, Chiang Mai, Thailand; TT Pham, DD Cuong, and HL Ha, Bach Mai Hospital, Hanoi, Vietnam; VK Nguyen, VH Bui, and TD Nguyen, National Hospital of Tropical Diseases, Hanoi, Vietnam; AH Sohn, N Durier and B Petersen, TREAT Asia, amfAR - The Foundation for AIDS Research, Bangkok, Thailand; DA Cooper, MG Law, A Jiamsakul and DC Boettiger, The Kirby Institute, The University of New South Wales, Sydney, Australia (TAHOD Steering Committee member; † Steering Committee Chair). **TREAT Asia Pediatric HIV Observational Database (TApHOD):** DK Wati, LPP Atmikasari, and IY Malino, Sanglah Hospital, Udayana University, Bali, Indonesia; R Nallusamy†, and KC Chan, Penang Hospital, Penang, Malaysia; P Lumbiganon, P Kosalaraksa, P Tharnprisan, and T Udomphanit, Khon Kaen University, Khon Kaen, Thailand; K Chokephaibulkit, K Lapphra, W Phongsamart, and O Wittawatmongkol, Siriraj Hospital, Mahidol University, Bangkok, Thailand; KTK Dung, NV Lam, PN An, and NT Loan, National Hospital of Pediatrics, Hanoi, Vietnam; HK Truong, TQ Du, and NH Chau, Children’s Hospital 1, Ho Chi Minh City, Vietnam; CV Do, and MT Ha, Children’s Hospital 2, Ho Chi Minh City, Vietnam; AH Sohn, N Durier, and P Nipathakosol, TREAT Asia, amfAR - The Foundation for AIDS Research, Bangkok, Thailand; DA Cooper, MG Law, and A Kariminia, The Kirby Institute, University of New South Wales, Sydney, Australia (TApHOD Steering Committee member; † Steering Committee Chair).

**The IeDEA Central Africa region**: Jean Claude Dusingize and Eugene Mutimura, Andre Gitembagara (Women’s Equity in Access to Care and Treatment, Kigali Rwanda); Kathryn Anastos (Albert Einstein College of Medicine and Montefiore Medical Center, New York, USA); Judy Tatwangire, Izimukwiye Izabelle, (Rwanda Military Hospital, Kigali, Rwanda); Theodore Niyongabo, Christelle Twizere(Centre hospitalo-Universitaire de Kamenge, Bujumbura, Burundi); Evelyne Baramperanye, (Centre National de Reference en matiere du VIH, Bujumbura ,Burundi); Andrew Edmonds, Marcel Yotebieng (Kalembelembe Pediatric hospital, Kinshasa, DRC); Innocent Azinyue, Liliane Ayangma, (Military Hospital of Yaoundé).

**The IeDEA East Africa region:** We acknowledge the contributions of the East African IeDEA sites to this analysis and would like to thank Dr. Ayaya at the The Academic Model Providing Access To Healthcare (AMPATH) Program, Eldoret Kenya ; Drs. Bukusi, Cohen, and Ms. Kulzer at Family AIDS Care and Education Services (FACES), Kisumu, Kenya ; National AIDS Control Programme in Tanzania (PI, Dr. Geoffrey Somi) Morogoro Regional Hospital (PI, Dr. Rita Lyamuya),Tumbi Regional Hospital (PI, Dr. Kapella Ngonyani), Masaka Regional Referral Hospital ( Dr. John Ssali and Mr. Haruna Ssemuwemba), Mbarara University of Science and Technology (MUST) ISS Clinic ( Dr. Bosco Bwana Mwebesa), Infectious Diseases Institute, Mulago ( Dr. Andrew Kambugu and Dr. Serbine Hermans) and Rakai Health Sciences Program ( Dr. Fred Nalugoda and Dr. Kasule Jjingo).

**The IeDEA Southern Africa region:** Member Sites: Cleophas Chimbetete, Newlands Clinic, Harare, Zimbabwe ; Diana Dickinson, Gabarone Independent, Botswana ; Brian Eley, Red Cross Children's Hospital, Cape Town, South Africa ; Christiane Fritz, SolidarMed Zimbabwe, Zimbabwe; Daniele Garone, Khayelitsha ART Programme and Médecins Sans Frontières, Cape Town, South Africa ; Janet Giddy, McCord Hospital, Durban, South Africa ; Christopher Hoffmann, Aurum Institute for Health Research, South Africa; Patrick MacPhail, Themba Lethu Clinic, Helen Joseph Hospital, Johannesburg, South Africa ; Harry Moultrie, Wits Institute for Sexual Reproductive Health, HIV & Related Diseases, Faculty of Health Sciences, University of the Witwatersrand, Johannesburg, and Harriet Shezi Children’s Clinic, Chris Hani Baragwanath Hospital, Soweto, South Africa; James Ndirangu, Hlabisa HIV Treatment and Care Programme, South Africa; Sabrina Pestilli, SolidarMed Mozambique, Mozambique; Sam Phiri, Lighthouse Clinic, Lilongwe, Malawi ; Hans Prozesky and Helena Rabie, Tygerberg Academic Hospital, Cape Town, and Pediatrics and Child Health, University of Stellenbosch, South Africa ; Jeff Stringer, Center for Infectious Disease Research in Zambia, Zambia ; Karl Technau, Empilweni Service and Research Unit, Rahima Moosa Mother and Child Hospital, University of the Witwatersrand, Johannesburg, South Africa; Robin Wood, Gugulethu and Masiphumelele ART Programmes and Desmond Tutu HIV Centre, Cape Town, South Africa . Central Team: Matthias Egger, Claire Graber, Fritz Kaeser, Olivia Keiser, Institute of Social and Preventive Medicine, University of Bern, Bern, Switzerland; Andrew Boulle, Morna Cornell, Mary-Ann Davies, Nicola Maxwell, School of Public Health and Family Medicine, University of Cape Town, Cape Town, South Africa.

**The IeDEA West Africa region:** Participating sites (*members of the Steering Committee, ^§^members of the Executive Committee): *Benin, Cotonou:* Adults: Djimon Marcel Zannou*, Carin Ahouada, Jocelyn Akakpo, Christelle Ahomadegbé, Jules Bashi, Alice Gougounon-Houéto, Angèle Azon-Kouanou, Fabien Houngbé, Jean Sehonou (CNHU Hubert Maga). Pediatrics: Sikiratou Koumakpaï*^§^, Florence Alihonou, Marcelline d’Almeida, Irvine Hodonou, Ghislaine Hounhoui, Gracien Sagbo, Leïla Tossa-Bagnan, Herman Adjide (CNHU Hubert Maga). ***Burkina Faso:*** Adults: Joseph Drabo*, René Bognounou, Arnaud Dienderé, Eliezer Traore, Lassane Zoungrana, Béatrice Zerbo (CHU Yalgado*,* ***Ouagadougou***), Adrien Bruno Sawadogo*^§^, Jacques Zoungrana, Arsène Héma, Ibrahim Soré, Guillaume Bado, Achille Tapsoba (CHU Souro Sanou, ***Bobo Dioulasso***) Pediatrics: Diarra Yé*, Fla Kouéta, Sylvie Ouedraogo, Rasmata Ouédraogo, William Hiembo, Mady Gansonré (CH Charles de Gaulle*,* ***Ouagadougou***). ***Côte d’Ivoire, Abidjan:*** Adults: Eugène Messou*, Joachim Charles Gnokoro, Mamadou Koné, Guillaume Martial Kouakou, (ACONDA-CePReF); Clarisse Amani Bosse*, Kouakou Brou, Achi Isidore Assi (ACONDA-MTCT-Plus); Henri Chenal*, Denise Hawerlander, Franck Soppi (CIRBA); Albert Minga*, Yao Abo, Jean-Michel Yoboue (CMSDS/CNTS); Serge Paul Eholié*^§^, Mensah Deborah Noelly Amego, Viviane Andavi, Zelica Diallo, Frédéric Ello, Aristophane Koffi Tanon (SMIT, CHU de Treichville), Serge Olivier Koule*, Koffi Charles Anzan, Calixte Guehi (USAC, CHU de Treichville). Pediatrics: Edmond Addi Aka*, Koffi Ladji Issouf, Jean-Claude Kouakou, Marie-Sylvie N’Gbeche, (ACONDA-CePReF); Touré Pety*, Divine Avit-Edi (ACONDA-MTCT-Plus); Kouadio Kouakou*, Magloire Moh, Valérie Andoblé Yao (CIRBA); Madeleine Amorissani Folquet*, Marie-Evelyne Dainguy, Cyrille Kouakou, Véronique Tanoh Méa-Assande, Gladys Oka-Berete, Nathalie Zobo, Patrick Acquah, Marie-Berthe Kokora (CHU Cocody); Tanoh François Eboua*, Marguerite Timité-Konan, Lucrèce Diecket Ahoussou, Julie Kebé Assouan, Mabéa Flora Sami, Clémence Kouadio (CHU Yopougon). ***Ghana, Accra:*** Pediatrics: Lorna Renner*^§^, Bamenla Goka, Jennifer Welbeck, Adziri Sackey, Seth Ntiri Owiafe (Korle Bu TH). ***Guinea-Bissau:*** Adults: Christian Wejse*^§^, Zacarias José Da Silva*, Joao Paulo (Bandim Health Project), The Bissau HIV cohort study group: Amabelia Rodrigues (Bandim Health Project), David da Silva (National HIV program Bissau), Candida Medina (Hospital National Simao Mendes, Bissau), Ines Oliviera-Souto (Bandim Health Project), Lars Østergaard (Dept of Infectious Diseases, Aarhus University Hospital), Alex Laursen (Dept of Infectious Diseases, Aarhus University Hospital), Morten Sodemann (Dept of Infectious Diseases, Odense University Hospital), Peter Aaby (Bandim Health Project), Anders Fomsgaard (Dept. of Virology, Statens Serum Institut, Copenhagen), Christian Erikstrup (Dept. of Clinical Immunology), Jesper Eugen-Olsen (Dept. of Infectious Diseases, Hvidovre Hospital, Copenhagen). ***Mali, Bamako:*** Adults: Moussa Y Maïga*^§^, Fatoumata Fofana Diakité, Abdoulaye Kalle, Drissa Katile (CH Gabriel Toure), Hamar Alassane Traore*, Daouda Minta*, Tidiani Cissé, Mamadou Dembelé, Mohammed Doumbia, Mahamadou Fomba, Assétou Soukho Kaya, Abdoulaye M Traoré, Hamady Traoré, Amadou Abathina Toure (CH Point G). Pediatrics: Fatoumata Dicko*, Mariam Sylla, Alima Berthé, Hadizatou Coulibaly Traoré, Anta Koïta, Niaboula Koné, Clémentine N'Diaye, Safiatou Touré Coulibaly, Mamadou Traoré, Naïchata Traoré (CH Gabriel Toure). ***Nigeria:*** Adults: Man Charurat* (UMB/IHV), Samuel Ajayi*, Georgina Alim, Stephen Dapiap, Otu (UATH, ***Abuja***), Festus Igbinoba (National Hospital ***Abuja***), Okwara Benson*, Clément Adebamowo*, Jesse James, Obaseki, Philip Osakede (UBTH, ***Benin*** ***City***), John Olasode (OATH, ***Ile-Ife***). ***Senegal, Dakar:*** Adults: Moussa Seydi*, Papa Salif Sow, Bernard Diop, Noël Magloire Manga, Judicael Malick Tine^§^, Coumba Cissé Bassabi (SMIT, CHU Fann), Pediatrics: Haby Signate Sy*, Abou Ba, Aida Diagne, Hélène Dior, Malick Faye, Ramatoulaye Diagne Gueye, Aminata Diack Mbaye (CH Albert Royer). ***Togo, Lomé:*** Adults: Akessiwe Patassi*, Awèrou Kotosso, Benjamin Goilibe Kariyare, Gafarou Gbadamassi, Agbo Komi, Kankoé Edem Mensah-Zukong, Pinuwe Pakpame (CHU Tokoin/Sylvanus Olympio). Pediatrics: Annette Koko Lawson-Evi*^§^, Yawo Atakouma, Elom Takassi, Améyo Djeha, Ayoko Ephoévi-gah, Sherifa El-Hadj Djibril (CHU Tokoin/Sylvanus Olympio). **Executive Committee*:** François Dabis (Principal Investigator, Bordeaux, France), Emmanuel Bissagnene (Co-Principal Investigator, Abidjan, Côte d’Ivoire), Elise Arrivé (Bordeaux, France), Patrick Coffie (Abidjan, Côte d’Ivoire), Didier Ekouevi (Abidjan, Côte d’Ivoire), Antoine Jaquet (Bordeaux, France), Valériane Leroy (Bordeaux, France), Charlotte Lewden (Bordeaux, France), Annie J Sasco (Bordeaux, France). **Operational and Statistical Team:** Dieudonné Amani (Abidjan, Côte d’Ivoire), Jean-Claude Azani (Abidjan, Côte d’Ivoire), Eric Balestre (Bordeaux, France), Serge Bessekon (Abidjan, Côte d’Ivoire), Franck Bohossou (Abidjan, Côte d’Ivoire), Camille Gilbert (Bordeaux, France), Sophie Karcher (Bordeaux, France), Jules Mahan Gonsan (Abidjan, Côte d’Ivoire), Jérôme Le Carrou (Bordeaux, France), Séverin Lenaud (Abidjan, Côte d’Ivoire), Célestin Nchot (Abidjan, Côte d’Ivoire), Karen Malateste (Bordeaux, France), Amon Roseamonde Yao (Abidjan, Côte d’Ivoire), Bertine Siloué (Abidjan, Côte d’Ivoire). **Administrative Team:** Gwenaelle Clouet (Bordeaux, France), Madikona Dosso (Abidjan, Côte d’Ivoire), Alexandra Doring^§^ (Bordeaux, France), Adrienne Kouakou (Abidjan, Côte d’Ivoire), Elodie Rabourdin (Bordeaux, France), Jean Rivenc (Pessac, France). **Consultants/ Working Groups:** Xavier Anglaret (Bordeaux, France), Boubacar Ba (Bamako, Mali), Jean Bosco Essanin (Abidjan), Andrea Ciaranello (Boston, USA), Sébastien Datté (Abidjan, Côte d’Ivoire), Sophie Desmonde (Bordeaux, France), Jean-Serge Elvis Diby (Abidjan, Côte d’Ivoire), Geoffrey S.Gottlieb* (Seattle, USA), Apollinaire Gninlgninrin Horo (Abidjan, Côte d’Ivoire), Serge N'zoré Kangah (Abidjan, Côte d’Ivoire), Denis Malvy (Bordeaux, France), David Meless (Abidjan, Côte d’Ivoire) , Aida Mounkaila-Harouna (Bordeaux, France), Camille Ndondoki (Bordeaux, France), Caroline Shiboski (San Francisco USA), Boris Tchounga (Abidjan, Côte d’Ivoire), Rodolphe Thiébaut (Bordeaux, France), Gilles Wandeler (Dakar, Senegal). **Coordinating Centre:** ISPED, Univ Bordeaux Segalen, Bordeaux, France **Regional Office:** PAC-CI, Abidjan, Côte d’Ivoire **Methodologic Support**: MEREVA, Bordeaux, France **Website:** http://www.mereva.net/iedea.

**The IeDEA Caribbean, Central and South America region**: Primary investigators: Catherine McGowan (Vanderbilt University, USA), Pedro Cahn (Fundación Huésped, Argentina), Eduardo Gotuzzo (Instituto de Medicina Tropical Alexander von Humboldt, UPCH, Perú), Marcelo Wolff Reyes (Fundación Arriarán, Universidad de Chile, Chile), Beatriz Grinsztejn (Fiocruz - Instituto de Pesquisa Clínica Evandro Chagas, Brazil), Jean William Pape (Les Centres GHESKIO, Haiti), Denis Padgett (Instituto Hondureño de Seguridad Social and Hospital Escuela, Honduras), Juan Sierra Madero (Instituto Nacional de Ciencias Médicas y Nutrición Salvador Zubirán, Mexico).
