# Supplementary material for: Implementation of Tuberculosis Intensive Case Finding, Isoniazid Preventive Therapy, and Infection Control ("Three I's") and HIV-Tuberculosis Service Integration in Lower Income Countries
Source: PLoS One. 2016 Apr 13;11(4):e0153243. doi: 10.1371/journal.pone.0153243 (PMC4830552; doi:10.1371/journal.pone.0153243)
Supplement: S2 File — (DOC) [file pone.0153243.s002.doc]

**Supplemental File S2:** Ethics statement including a full list of Ethics Committees and/or Institutional Review Boards.

Data were collected through IeDEA Cohorts. Ethics committees and/or institutional review boards in all host countries approved the collection and transfer of anonymized data, namely: Beijing Ditan Hospital IRB, Beijing, China; Kerti Praja Foundation IRB, Denpasar, Bali, Indonesia; Ministry of Health Malaysia IRB, Selangor, Malaysia; Research Institute for Tropical Medicine IRB, Department of Health, Muntinlupa, Metro Manila, Philippines; Chulalongkorn University IRB, Bangkok, Thailand; Khon Kaen University Ethics Committee for Human Research, Khon Kaen, Thailand; Faculty of Medicine, Chiang Mai University IRB, Chiang Mai, Thailand; Siriraj Hospital, Mahidol University, Bangkok, Thailand; Ramathibodi Hospital, Mahidol University, Bangkok, Thailand; National Hospital for Tropical Diseases IRB, Hanoi, Vietnam; Hanoi School of Public Health IRB, Hanoi, Vietnam; Children's Hospital 1 IRB, Ho Chi Minh City, Vietnam; Comite de Bioética Fundación Huésped IRB #1, Argentina; Comite - CEP IPEC, Brazil; Servicio de Salud Metropolitano Central IRB #1, Chile; Gheskio Ctrs IRB #1- Comite des Droits Humains, Haiti ; Unidad de Investigación Cientifica IRB #1, Honduras; Instituto Nacional de Ciencias Médicas y Nutrición Salvador Zubiran IRB #1, Mexico; Universidad Peruana Cayetano Heredia IRB #1, Lima, Peru; Comité National d'Ethique au Burundi ,Bujumbura, Burundi ; Cameroon National Ethics, Yaounde, Cameroon; Rwanda National Ethics Committee, Kigali Rwanda; Moi University College of Health Sciences (MU/CHS) & Moi Teaching and Referral Hospital (MT&RH) Institutional Research and Ethics Committee (IREC); Kenya Medical Research Institute/National Ethics Review Committee (ERC); The United Republic of Tanzania National Institute for Medical Research Coordinating Committee; The United Republic of Tanzania National Institute for Medical Research Coordinating Committee; Mbarara University of Science & Technology Institutional Review Committee (MUST-IRC); Makerere University School Medicine Research & Ethics Committee (MUSOMREC); Uganda Virus Research Institute Science & Ethics Committee (UVRI-SEC); Indiana University Institutional Review Board; Hlth Rsch Unit IRB #1, Gaborone, Botswana; National Hlth Sci Rsch Committee IRB #1, Lilongwe, Malawi; U of Cape Town IRB #1 - Senate Ethics in Rsch Committee, South Africa; Human Research Ethics Committee (Medical), University of Witwatersrand , Johannesburg, South Africa; U of Stellenbosch IRB #2 - Human Rsch, Cape Town South Africa; U of Zambia, Rsch Ethics Committee IRB #1, Lusaka, Zambia; Medical Rsch Council Zimbabwe IRB #1, Harare, Zimbabwe; Ministère de la Santé IRB #1 – CNPERS, Cotonou, Benin; Centre Natl de Recherche et de Formation sur le Paludism IRB #1 - CERS Ouagadougou, Burkina Faso; Comité National d'Ethique et de la Recherche (CNER) IRB #1, Abidjan, Côte d'Ivoire; U of Abuja Teaching Hosp IRB #1, Abuja, FCT, NIGERIA; U Benin Teaching Hosp IRB #1 Benin City, Nigeria; Conseil Natl de Recherche En Sante frmly Direction des Etudes IRB #1, Dakar, Senegal ; Kantonale Ethikkommission Bern, Switzerland; Health Sciences Faculty research Ethics Committee, University of Cape Town, South Africa. Where requested per local regulations informed consent was provided. In addition, the Vanderbilt University Health Science Committee, Nashville, Tennessee (USA), the Ethics Committee of the University of Bern (Switzerland), and the University of Cape Town (South Africa) approved the analyses of these observational data for this specific project.
